# Supplementary material for: Long-Distance Movement of Solanum tuberosum Translationally Controlled Tumor Protein (StTCTP) mRNA
Source: Plants (Basel). 2023 Aug 1;12(15):2839. doi: 10.3390/plants12152839 (PMC10420919; doi:10.3390/plants12152839)
Supplement: Supplementary file 1 [file plants-12-02839-s001.zip › plants-2484132-supplementary.pdf]

Supplementary Material

Supplementary figures

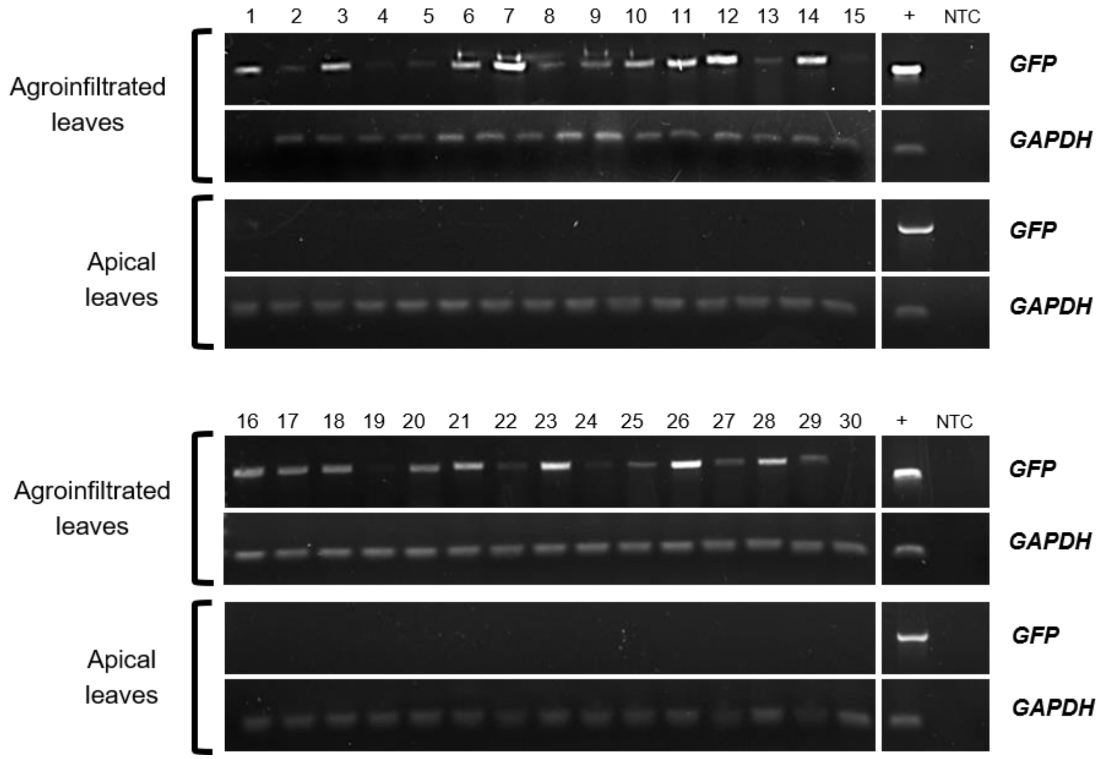

Figure S1. *GFP* and *GAPDH* mRNA detection in agroinfiltrated and apical leaves from potato plants transiently transformed with *35S::GFP-GUS* construct. Agarose gel electrophoresis of endpoint RT-PCR products from total RNA of transiently transformed plants (1-30). *GFP*: green fluorescent protein, reporter gene (720 bp). *GAPDH*: glyceraldehyde-3-phosphate dehydrogenase, endogenous gene (92 bp). +: positive control. NTC: non-template control.

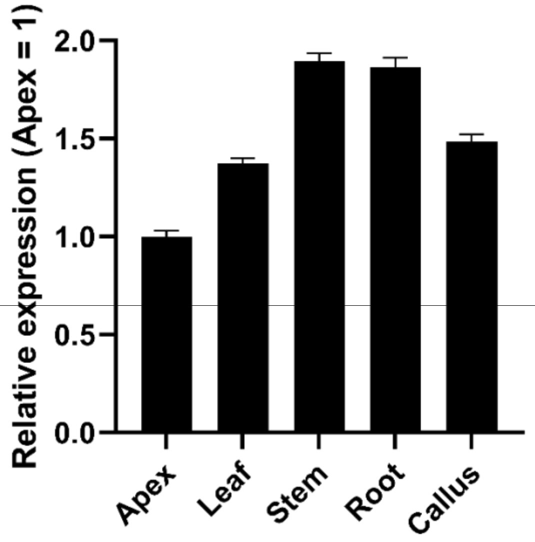

Figure S2. Endogenous *StTCTP* transcript levels. Endogenous *StTCTP* mRNA was detected by quantitative RT-PCR in apex, leaf, stem, root and callus samples. *GAPDH* was used as endogenous control. Mean of three biological samples in triplicate is shown. Relative expression was calculated with Apex = 1. Bars represent standard error of the mean.

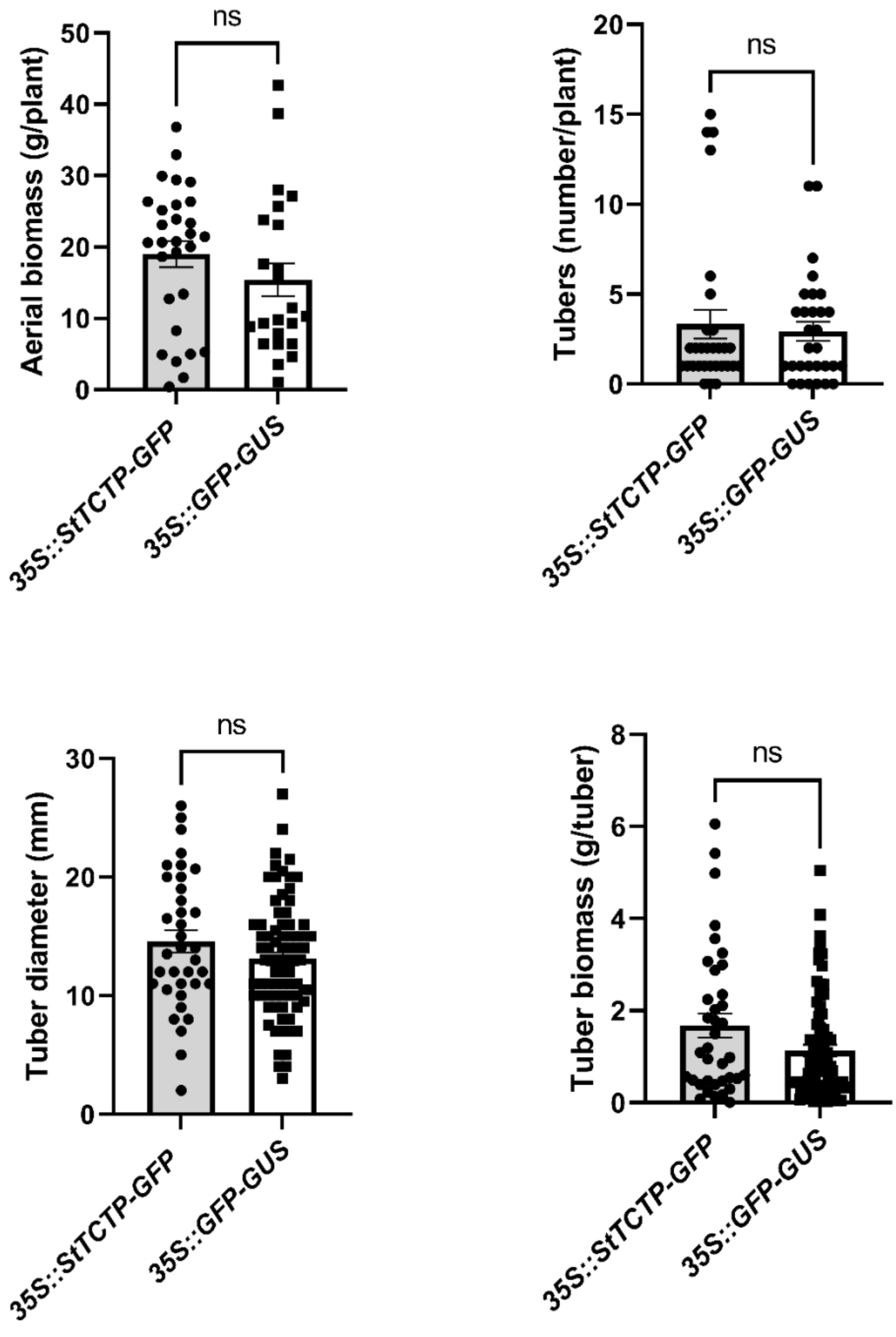

Figure S3. Phenotype of agroinfiltrated plants with *35S::StTCTP-GFP* or *GFP::GUS* constructions. Plants were analyzed 40 days after agroinfiltration with *35S::StTCTP-GFP* or *35S::GFP-GUS* vectors, dispersion is represented with black circles and squares, respectively. *35S::StTCTP-GFP* n=30; *35S::GFP-GUS* n= 30. Bars represent standard error of the mean. Asterisks symbolize significant difference according to Mann Whitney tests: not significant (ns).

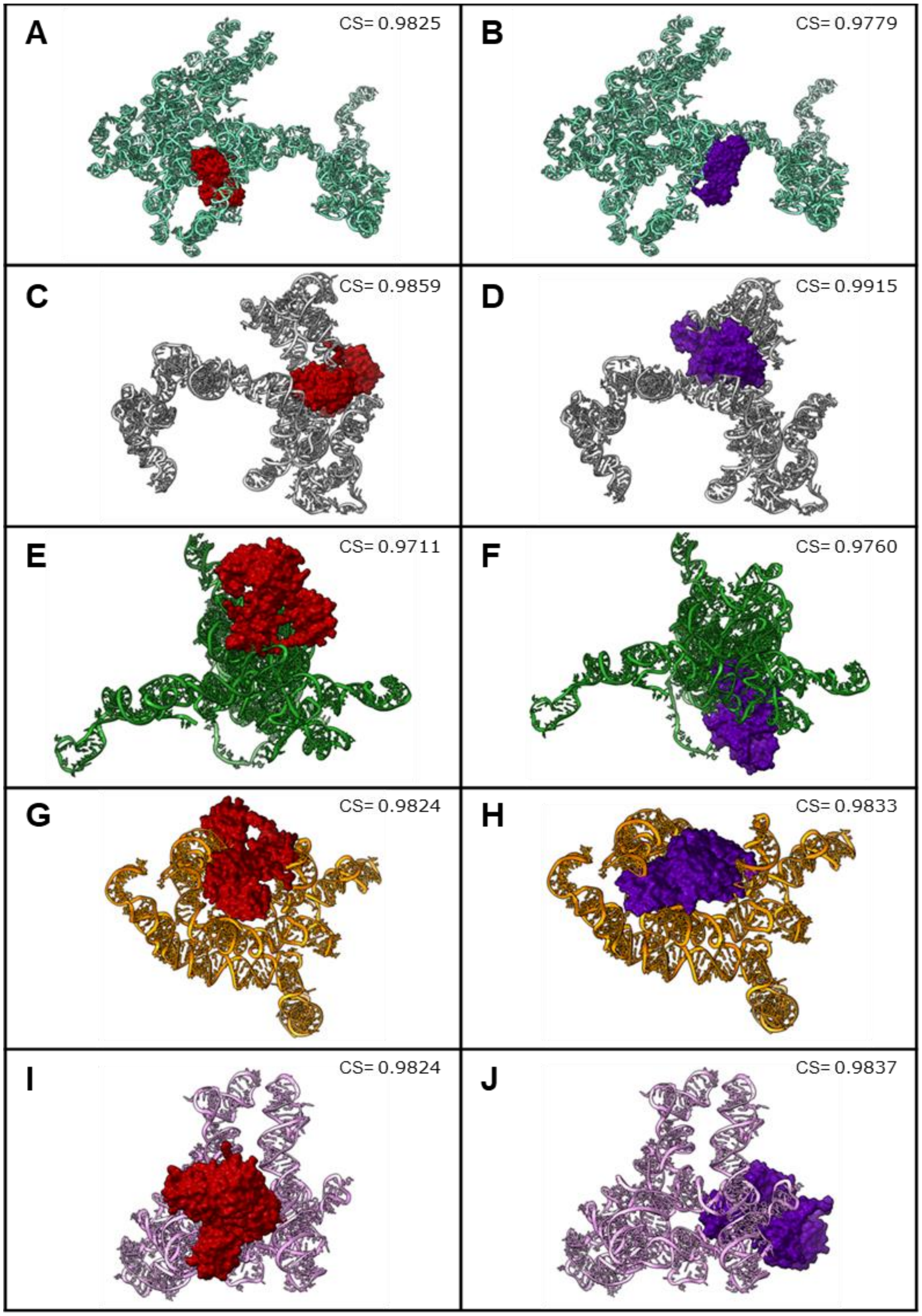

Figure S4. *In silico* docking analysis of *TCTP* transcripts and PTB1/6 proteins. Hypothesized 3D structures of *StTCTP*, *CmTCTP*, *AtTCTP1*, *AtTCTP2*, and *StBEL5* transcripts were obtained. Likewise, the predictions of the PTB1 and PTB6 proteins were obtained, which were refined. The 3D coupling of each protein with the structures of the transcripts was calculated independently. Red and purple structures correspond to PTB1 and PTB6, respectively in all cases. The couplings between PTB1 and PTB6 with the 3D structure of *StBEL5* mRNA (A and B), *CmTCTP* mRNA (C and D), *AtTCTP1* mRNA (E and F), *AtTCTP2* mRNA (G and H), and *StTCTP* mRNA (I and J) are shown. Confidence scores (SC) were calculated in HDOCK.

Supplementary tables

Table S1. Number of tubers obtained from plants agroinfiltrated with *35S::StTCTP-GFP* or *35S::GFP-GUS* vectors.

**Vector**	**Tuberization stages (Tuber number/stage)**								
	**I**	**II**	**III**	**IV**	**V**	**VI**	**VII**	**VIII**	**Total**
35S::StTCTP-GFP	0	2	5	4	1	1	2	3	18
	2	2	1	2	2	3	2	2	16
	0	0	0	0	0	0	0	15	15
	0	0	0	0	0	0	0	14	14

0	0	0	0	0	0	0	14	14
2	0	0	2	4	2	2	1	13
0	0	0	0	0	0	0	13	13
1	2	0	2	1	1	2	2	11
2	1	0	0	2	1	2	3	11
1	0	2	0	3	1	0	2	9
0	1	2	1	1	1	1	2	9
0	0	1	0	6	0	0	2	9

0	0	0	0	0	0	0	6	6
0	0	0	0	0	0	0	5	5
0	0	0	0	0	0	0	2	2
0	0	0	0	0	0	0	2	2
0	0	0	0	0	0	0	2	2
0	0	0	0	0	0	0	1	1
0	0	0	0	0	0	0	1	1
0	0	0	0	0	0	0	1	1

0	0	0	0	0	0	0	1	1
0	0	0	0	0	0	0	1	1
0	0	0	0	0	0	0	1	1
0	0	0	0	0	0	0	1	1
0	0	0	0	0	0	0	1	1
0	0	0	0	0	0	0	1	1
0	0	0	0	0	0	0	1	1
0	0	0	0	0	0	0	0	0

	0	0	0	0	0	0	0	0	0
	0	0	0	0	0	0	0	0	0
**Total**	8	8	11	11	20	10	11	100	**179**
	0	0	0	0	0	0	0	11	11
	0	0	0	0	0	0	0	11	11
35S::GFP-GUS	0	0	0	0	0	0	0	7	7
	0	0	0	0	0	0	0	6	6
	0	0	0	0	0	0	0	5	5

0	0	0	0	0	0	0	5	5
0	0	0	0	0	0	0	5	5
0	0	0	0	0	0	0	4	4
0	0	0	0	0	0	0	4	4
0	0	0	0	0	0	0	4	4
0	0	0	0	0	0	0	4	4
0	0	0	0	0	0	0	4	4
0	0	0	0	0	0	0	3	3

	0	0	0	0	0	0	0	3	3
	0	0	0	0	0	0	0	2	2
	0	0	0	0	0	0	0	2	2
	0	0	0	0	0	0	0	1	1
	0	0	0	0	0	0	0	1	1
	0	0	0	0	0	0	0	1	1
	0	0	0	0	0	0	0	1	1
	0	0	0	0	0	0	0	1	1

0	0	0	0	0	0	0	1	1
0	0	0	0	0	0	0	1	1
0	0	0	0	0	0	0	1	1
0	0	0	0	0	0	0	0	0
0	0	0	0	0	0	0	0	0
0	0	0	0	0	0	0	0	0
0	0	0	0	0	0	0	0	0
0	0	0	0	0	0	0	0	0

	0	0	0	0	0	0	0	0	0
**Total**	0	0	0	0	0	0	0	88	**88**

* Information of the tuber number per plant of *35S::StTCTP-GFP* (n=30) and *35S::GFP-GUS* (n=30) vectors is shown.

Table S2. MolProbity score calculation of 3D mRNA models.

mRNA	MolProbity parameters	Model 1	Model 2	Model 3	Model 4	Model 5
StBEL5 [1]	Probably wrong sugar puckers	752	754	749	722	729

	Bad backbone conformations	1245	1229	1213	1203	1199
	Bad bonds	391 / 48892	413 / 48892	482 / 48892	488 / 48890	559 / 48891
	Bad angles	3170 / 76148	3423 / 76148	3612 / 76148	3537 / 76140	3816 / 76144
	Chiral handedness swaps	1321/8212	1242/8212	1272/8212	1124/8212	1156/8212
	Tetrahedral geometry outliers	25	32	43	31	56
StTCTP [2]	Probably wrong sugar puckers	149	175	172	171	184

	Bad backbone conformations	253	305	286	295	293
	Bad bonds	0 / 12895	41 / 12895	22 / 12895	1 / 12895	31 / 12894
	Bad angles	376 / 20090	608 / 20090	493 / 20090	385 / 20090	567 / 20086
	Chiral handedness swaps	265/2164	336/2164	302/2164	352/2164	360/2164
	Tetrahedral geometry outliers	1	1	0	0	1
AtTCTP1 [3]	Probably wrong sugar puckers	258	239	263	254	256

	Bad backbone conformations	258	414	432	441	438
	Bad bonds	32 / 19026	12 / 19026	53 / 19026	14 / 19026	41 / 19026
	Bad angles	916 / 29626	619 / 29626	939 / 29626	659 / 29626	861 / 29626
	Chiral handedness swaps	515/3216	467/3216	492/3216	517/3216	517/3216
	Tetrahedral geometry outliers	7	0	5	2	2
AtTCTP2 [4]	Probably wrong sugar puckers	158	176	168	163	168

	Bad backbone conformations	272	280	280	271	263
	Bad bonds	27 / 12112	27 / 12112	21 / 12112	1 / 12112	21 / 12112
	Bad angles	587 / 18883	515 / 18883	562 / 18883	389 / 18883	541 / 18883
	Chiral handedness swaps	286/2020	316/2020	328/2020	308/2020	280/2020
	Tetrahedral geometry outliers	2	2	1	2	5
CmTCTP [5]	Probably wrong sugar puckers	261	263	235	269	275

Bad backbone conformations	448	453	407	448	454
Bad bonds	118 / 19045	75 / 19045	15 / 19045	95 / 19045	82 / 19045
Bad angles	1139 / 29673	989 / 29673	646 / 29673	1037 / 29673	1014 / 29673
Chiral handedness swaps	424/3200	482/3200	412/3200	482/3200	465/3200
Tetrahedral geometry outliers	5	3	0	1	4

[1] Model 2 was selected for *StBEL5*
[2] Model 1 was selected for *StTCTP*
[3] Model 2 was selected for *AtTCTP1*
[4] Model 4 was selected for *AtTCTP2*
[5] Model 3 was selected for *CmTCTP*

Table S3. mRNA Structure Validation: Pseudotorsion Plots.

mRNA	Torsion angles	Model 1	Model 2	Model 3	Model 4	Model 5
StBEL5 [1]	C3' Eta-Theta	5.31 %	5.38%	5.95 %	5.87 %	5.33
	C2' Eta-Theta	15.67 %	12.95%	16.74 %	14.71 %	14.29 %
	C3' Eta′-Theta′	4.55 %	3.95%	4.84 %	4.93 %	3.93 %
	C2' Eta′-Theta′	36.19 %	37.95%	34.42 %	34.8 %	39.43 %
	C3' Eta′′-Theta′′	3.38 %	3.00%	3.67 %	3.94 %	3.74 %
	C2' Eta′′-Theta′′	10.45 %	8.93%	6.05 %	13.24 %	9.14 %

	C3' Eta-Theta	4.09%	2.3 %	3.48 %	3.1 %	4.46 %
	C2' Eta-Theta	8.06%	12.9 %	7.02 %	7.22 %	14.75 %
	C3' Eta′-Theta′	2.16%	2.3 %	2.74 %	2.33 %	2.36 %
StTCTP [2]	C2' Eta′-Theta′	37.10%	40.32 %	42.11 %	32.99 %	42.62 %
	C3' Eta′′-Theta′′	2.64%	2.56 %	2.49 %	3.62 %	3.15 %
	C2' Eta′′-Theta′′	11.29%	4.84 %	7.02 %	7.22 %	16.39 %
AtTCTP1 [3]	C3' Eta-Theta	3.58 %	4.04%	2.55 %	3.63 %	4.06 %

	C2' Eta-Theta	10.59 %	9.92%	18.42 %	13.14 %	16.88 %
	C3' Eta′-Theta′	3.41 %	3.20%	2.72 %	2.77 %	3.38 %
	C2' Eta′-Theta′	48.24 %	31.40%	51.32 %	37.96 %	31.17 %
	C3' Eta′′-Theta′′	2.39 %	1.68%	2.04 %	1.21 %	1.52 %
	C2' Eta′′-Theta′′	4.71 %	4.13%	5.26 %	5.84 %	5.19 %
AtTCTP2 [4]	C3' Eta-Theta	3.73 %	5.74 %	4.48 %	3.02%	3.54 %
	C2' Eta-Theta	18.37 %	7.27 %	10.71 %	13.25%	14.04 %

	C3' Eta′-Theta′	2.67 %	4.37 %	3.36 %	2.75%	3.27 %
	C2' Eta′-Theta′	40.82 %	29.09 %	39.29 %	32.53%	40.35 %
	C3' Eta′′-Theta′′	1.87 %	3.55 %	2.24 %	2.20%	1.91 %
	C2' Eta′′-Theta′′	14.29 %	5.45 %	1.79 %	7.23%	10.53 %
	C3' Eta-Theta	4.07 %	2.77 %	7.87%	6.53 %	5.74 %
CmTCTP [5]	C2' Eta-Theta	15.73 %	11.22 %	10.20%	12.0 %	15.07 %
	C3' Eta′-Theta′	3.23 %	2.77 %	6.70%	5.41 %	5.41 %

C2' Eta′-Theta′	38.2 %	45.92 %	20.41%	14.0 %	9.59 %
C3' Eta′′-Theta′′	3.24 %	2.42 %	5.06%	3.84 %	4.24 %
C2' Eta′′-Theta′′	7.87 %	1.02 %	12.24%	14.0 %	6.85 %

[1] Model 2 was selected for *StBEL5*
[2] Model 1 was selected for *StTCTP*
[3] Model 2 was selected for *AtTCTP1*
[4] Model 4 was selected for *AtTCTP2*
[5] Model 3 was selected for *CmTCTP*

Table S4. MolProbity score calculation of PTB1 and PTB6 models.

Protein	MolProbity parameters	Model 1	Model 2	Model 3	Model 4	Model 5
PTB11	Poor rotamers	0	0	0	0	0
	Favored rotamers	385	385	385	385	385
	Ramachandran outliers	1	1	1	1	1
	Ramachandran favored	427	429	430	427	428
	Rama distribution Z-score	0.40 ± 0.41	0.51 ± 0.41	0.56 ± 0.40	0.49 ± 0.40	0.50 ± 0.40

Cβ deviations >0.25Å	0	0	0	0	0
Bad bonds:	0 / 3521	0 / 3521	0 / 3521	0 / 3521	0 / 3521
Bad angles:	12/4764	12/4764	12/4764	12/4764	12/4764
Cis Prolines:	0 / 19	0 / 19	0 / 19	0 / 19	0 / 19
Cis nonProlines:	2 / 421	2 / 421	2 / 421	2 / 421	2 / 421
CaBLAM outliers	11	12	11	11	11
CA Geometry outliers	7	6	6	6	6
Chiral volume outliers	0/525	0/525	0/525	0/525	0/525

	Poor rotamers	0	0	0	0	0
	Favored rotamers	383	383	384	384	384
	Ramachandran outliers	4	3	3	2	3
PTB6 2	Ramachandran favored	429	434	429	429	431
	Rama distribution Z-score	0.50 ± 0.39	0.61 ± 0.39	0.72 ± 0.40	0.97 ± 0.40	0.73 ± 0.41
	Cβ deviations >0.25Å	0	0	0	0	0
	Bad bonds:	0 / 3551	0 / 3551	0 / 3551	0 / 3551	0 / 3551

Bad angles:	12/4813	15 / 4813	14 / 4813	12/4813	14 / 4813
Cis Prolines:	0 / 22	0 / 22	0 / 22	0 / 22	0 / 22
Cis nonProlines:	2 / 421	2 / 421	2 / 421	2 / 421	2 / 421
CaBLAM outliers	5	4	8	9	5
CA Geometry outliers	6	5	5	5	7
Chiral volume outliers	0/530	0/530	0/530	0/530	0/530

[1] Model 3 was selected for PTB1
[2] Model 2 was selected for PTB6

Table S5. Structure validation score (SAVES v6.0) of PTB1 and PTB6 refined models.

Protein	Structure validation	Model 1	Model 2	Model 3	Model 4	Model 5
PTB1 [2]	ERRAT	95.619	95.90790	96.62340	96.63210	96.65810
	Verify 3D	85.26%	83.45%	84.35%	82.09%	84.58%

	Residues with averaged 3D-1D score >= 0.1					
	PROCHECK	Out of 9 evaluations Errors: 3 Warning: 3 Pass: 3	Out of 9 evaluations Errors: 3 Warning: 3 Pass: 3	Out of 9 evaluations Errors: 3 Warning: 3 Pass: 3	Out of 9 evaluations Errors: 3 Warning: 3 Pass: 3	Out of 9 evaluations Errors: 3 Warning: 3 Pass: 3
	RAMACHANDRAN Residues in most favored regions	90.8%	91%	91.3%	91.3%	91.3%
PTB6 [2]	ERRAT	95.0739	95.3771	94.7761	96.4377	94.321
	Verify 3D (Residues with averaged 3D-1D score >= 0.1)	86.26%	87.61%	85.59%	86.26%	84.46%
	PROCHECK	Out of 9 evaluations Errors: 3 Warning: 4	Out of 9 evaluations Errors: 3 Warning: 3	Out of 9 evaluations Errors: 3 Warning: 3	Out of 9 evaluations Errors: 3 Warning: 3	Out of 9 evaluations Errors: 3 Warning: 3

	Pass: 2	Pass: 3	Pass: 3	Pass: 3	Pass: 3
RAMACHANDRAN (Residues in most favored regions)	91%	92.3%	90.5%	92%	92%

[1] Model 3 was selected for PTB1
[2] Model 2 was selected for PTB6

Table S6. Quality of docking structures predicted in HDock.

Docking (Protein -mRNA)	Rank	Models									
		**1***	**2**	**3**	**4**	**5**	**6**	**7**	**8**	**9**	**10**
	Docking Score	-351.2	-309.0	-307.5	-284.9	-281.5	-279.5	-279.5	-278.4	-276.5	-276.1

PTB1-*StBEL5*	Confidence Score	0.9825	0.9601	0.959	0.937	0.9329	0.9303	0.9302	0.9289	0.9263	0.925
	Ligand rmsd (Å)	374.7	344.45	383.06	422.26	409.86	401.84	374.04	423.48	412.13	403.8
PTB1-*CmTCTP*	Docking Score	-362.4	-333.8	-325.1	-320.9	-312.3	-310.2	-308.3	-307.6	-305.2	-303.3
	Confidence Score	0.9859	0.9753	0.9708	0.9683	0.9626	0.961	0.9596	0.959	0.9571	0.955
	Ligand rmsd (Å)	180.4	164.95	147.76	119.29	131.41	138.3	130.88	139.56	145	156.6
PTB1-AtTCTP1	Docking Score	-325.7	-320.4	-315.1	-307.7	-296.3	-292.5	-291.5	-290.2	-288.2	-287.7
	Confidence Score	0.9711	0.968	0.9645	0.9591	0.9491	0.9454	0.9443	0.943	0.9407	0.940

	Ligand rmsd (Å)	238.3	216.16	158.81	137.06	214.91	205.58	132.05	147.86	184.81	154.9
PTB1-AtTCTP2	Docking Score	-351.0	-349.9	-346.7	-344.9	-342.6	-334.6	-327.2	-325.4	-324.4	-319.1
	Confidence Score	0.9824	0.982	0.9808	0.9801	0.9792	0.9757	0.9719	0.971	0.9704	0.967
	Ligand rmsd (Å)	103.23	104.04	89.9	144.88	116.3	73.7	68.9	130.55	109.65	65.87
PTB1-StTCTP	Docking Score	-327.8	-321.2	-320.7	-320.5	-319.7	-319.0	-315.1	-306.6	-302.8	-299.2
	Confidence Score	0.9723	0.9684	0.9682	0.9681	0.9676	0.9671	0.9645	0.9583	0.9551	0.951
	Ligand rmsd (Å)	173.1	126.63	125.54	138.71	124.49	165.66	133.33	108.91	140.94	110.3

PTB6-*BEL5*	Docking Score	-339.5	-315.7	-310.5	-291.8	-289.4	-288.5	-268.6	-263.1	-262.2	-262.2
	Confidence Score	0.9779	0.9649	0.9612	0.9446	0.9421	0.9411	0.9147	0.9058	0.9043	0.904
	Ligand rmsd (Å)	372.99	367.34	416.52	380.73	370.07	384.37	486.22	407.15	431.95	354.6
PTB6-*CmTCTP*	Docking Score	-387.9	-353.6	-349.9	-342.9	-342.0	-328.3	-322.0	-320.6	-319.6	-319.5
	Confidence Score	0.9915	0.9832	0.982	0.9794	0.979	0.9725	0.969	0.9681	0.9675	0.967
	Ligand rmsd (Å)	133.24	137.5	143.35	122.22	141.03	130.87	103.51	156.51	135.51	139.1
	Docking Score	-335.3	-333.9	-318.8	-315.5	-315.0	-312.4	-311.7	-305.5	-304.8	-301.2

PTB6-AtTCTP1	Confidence Score	0.976	0.9754	0.967	0.9648	0.9644	0.9627	0.9622	0.9573	0.9568	0.953
	Ligand rmsd (Å)	142.38	202.82	204.67	207.55	211	177.63	199.5	141.94	134.6	107.5
PTB6-AtTCTP2	Docking Score	-353.8	-347.4	-345.4	-335.8	-333.8	-329.9	-329.6	-328.7	-327.2	-326.8
	Confidence Score	0.9833	0.9811	0.9803	0.9763	0.9753	0.9734	0.9732	0.9728	0.9719	0.971
	Ligand rmsd (Å)	97	118.91	131.42	106.97	109.71	117.67	107.09	116.66	126.96	93.5
PTB6-StTCTP	Docking Score	-354.9	-354.6	-354.6	-338.4	-335.2	-317.8	-312.5	-311.9	-310.5	-309.3
	Confidence Score	0.9837	0.9836	0.9836	0.9775	0.976	0.9663	0.9627	0.9622	0.9613	0.960

Ligand rmsd (Å)	138.36	74.1	155.87	144.79	127.9	127.83	142.8	119.57	119.18	124.3

* Model 1 was selected for all docking protein-mRNA predictions

Table S7. List of oligonucleotide sequences.

Gen	Sense	Sequence (5′->3′)	Amplicon length (pb)

bar (RT-qPCR)	For	GTGCTTGTCTCGATGTAG	100
	Rev	GAGGGGATCTACCATGAG	
GAPDH (RT-qPCR and endpoint RT-PCR)	For	GCTGTTGGAAAGGTGCTACCATCATTG	92
	Rev	AGTGAGATCAACCACAGAGACATCGAC	
GFP (RT-qPCR)	For	ACTACAACAGCCACAACGTC	81
	Rev	TGTTGTGGCGGATCTTGAAG	
GFP ORF (Endpoint RT-PCR)	For	TTACTTGTACAGCTCGTCCATGCCGAG	720
	Rev	TCAATGGTGAGCAAGGGCGAGGAGCTGTT	

StTCTP (RT-qPCR)	For	TGCTTTGGGAAGTTCAAGGG	123
	Rev	ACAACCTTGACAGCTTGGTC	
StTCTP (PCR/Cloning)	For	ATGGCCCTTTCTTGGTATGGTGCTGTTCTTG	506
	Rev	CTAGCACTTGATCTCCTTCAAGCCAGGTGCAAGG	
e35S (PCR/Cloning)	For	CAACATGGTGGAGCACGACACTCTC	751
	Rev	TCAGCGTGTCCTCTCCAAATGAAATG	

Table S8. Accession numbers of mRNA sequences used for 3D predictions.

mRNA	Database	Accession number
StBEL5	NCBI [1]	XM_006361029.2
CmTCTP	NCBI [1]	DQ304537.2
StTCTP	Phytozome 13 [2]	PGSC0003DMT400063575
AtTCTP1	Phytozome 13 [2]	AT3G16640
AtTCTP2	Phytozome 13 [2]	AT3G05540

[1]https://www.ncbi.nlm.nih.gov/

[2] https://phytozome-next.jgi.doe.gov/
